# A Comparative Analysis of Visual Encoding Models Based on Classification and Segmentation Task-Driven CNNs

**DOI:** 10.1155/2020/5408942

**Published:** 2020-08-01

**Authors:** Ziya Yu, Chi Zhang, Linyuan Wang, Li Tong, Bin Yan

**Affiliations:** PLA Strategy Support Force Information Engineering University, Zhengzhou 450001, China

## Abstract

Nowadays, visual encoding models use convolution neural networks (CNNs) with outstanding performance in computer vision to simulate the process of human information processing. However, the prediction performances of encoding models will have differences based on different networks driven by different tasks. Here, the impact of network tasks on encoding models is studied. Using functional magnetic resonance imaging (fMRI) data, the features of natural visual stimulation are extracted using a segmentation network (FCN32s) and a classification network (VGG16) with different visual tasks but similar network structure. Then, using three sets of features, i.e., segmentation, classification, and fused features, the regularized orthogonal matching pursuit (ROMP) method is used to establish the linear mapping from features to voxel responses. The analysis results indicate that encoding models based on networks performing different tasks can effectively but differently predict stimulus-induced responses measured by fMRI. The prediction accuracy of the encoding model based on VGG is found to be significantly better than that of the model based on FCN in most voxels but similar to that of fused features. The comparative analysis demonstrates that the CNN performing the classification task is more similar to human visual processing than that performing the segmentation task.

## 1. Introduction

Complex neural circuits in the human brain allow us to easily understand the external visual world. However, the mechanisms of how visual areas encode visual stimuli have not yet been elucidated. Therefore, the development of a visual encoding model to predict the voxel response induced by any input stimulus, that is, simulating the complex nonlinear relationship between visual input and evoked voxel responses, has attracted wide attention [1, 2]. It can explain how the brain processes visual information through neural circuits [3]. In visual research based on functional magnetic resonance imaging (fMRI), linearized encoding has been widely applied to these models. It consists of a nonlinear mapping from visual stimuli to features and a linear mapping from features to voxel responses [4]. Nonlinear mapping is critical to visual encoding that can be implemented by various feature extractors such as Gabor wavelet pyramid (GWP) [5], histogram of oriented gradient (HOG) [6], local binary patterns (LBP) [7], scale-invariant feature transform (SIFT) [8], and convolution neural networks (CNNs). On the other hand, linear mapping generally uses linear regression models with specific regularization.

In recent years, CNNs have been widely used in visual encoding models. CNNs, proposed based on early discoveries of the network structure and the visual system [9], can be used in a variety of computer vision tasks such as image classification [10], target recognition [11], and semantic segmentation [12]. Studies have shown that a deep network is comparable to the human visual system, which can automatically learn effective features from large data for specific tasks and predict voxel responses measured by fMRI in a multilevel manner [13]. Agrawal et al. [14] first proposed a CNN to predict human brain activity based on low-level visual input (pixels). Güçlü and van Gerven [15, 16] illustrated the similarity between a CNN and the mechanism of visual processing in both the ventral visual pathway, which is responsible for object recognition, and the dorsal visual pathway, which is responsible for motion perception. These studies demonstrated that a CNN is similar to a visual pathway from a low level to a high level. Wen et al. [17] established an encoding model based on the deep residual network (DRN), which has been shown to perform better than the shallow AlexNet for video stimuli. Their study showed that improvements in prediction accuracy are due to the better feature expression of the deep network with a residual structure. Therefore, in computer vision, the choice of a network to obtain suitable feature transformations is critical, which directly influences the encoding performance [18].

In 2016, Yamins and DiCarlo [19] proposed a particularly important challenge, which was whether a model optimized for tasks other than classification can better explain neural data. In particular, task-driven deep networks performing different computer vision tasks can extract different features from the same image stimuli, resulting in variations in the performance of encoding models. Currently, studies on encoding models based on deep networks are limited to the visual classification task, which is different from the complexity and diversity of the human visual system.

Here, we explore the impact of network tasks on the performance of encoding models by building models based on the features extracted from a segmentation network, features extracted from a classification network, and the fusion of the two features. We use the largest dataset in the published dataset, BOLD5000 [20], to train and test the encoding model. We calculate the Pearson Correlation Coefficient between the predicted and experimental fMRI responses to compare the prediction performances of the three encoding models. Using the results, we describe the impact of changes in network tasks on the visual encoding model. We then discuss the advantages and disadvantages of simulating the human visual processing.

In this study, our main contributions are as follows: (1) we analyze the drawbacks of current encoding methods based on the complexity and diversity of the human visual system, (2) we propose to employ different task-driven networks to construct encoding models, and (3) we analyze the impact of different task-driven networks on the performance of encoding models and provide a possible direction for subsequent research on visual encoding.

## 2. Materials and Methods

### 2.1. Experimental Data

We used the public fMRI dataset, BOLD5000 [20], which can be downloaded from https://bold5000.github.io/download.html. Details of the visual stimuli and fMRI protocols of the dataset have been discussed elsewhere [20]. Hence, we only briefly summarize the details of the dataset in this subsection.

The dataset comprised fMRI data collected from four subjects, with three having a full set of data. Hence, we only used the data of three subjects. A full set of data included 16 MRI scan sessions, with 15 functional sessions and a session for the acquisition of high-resolution anatomical and diffusion data. Each functional session lasted 1.5 hours, consisting of 8 sessions with 9 image runs and an additional functional localizer run and 7 sessions with 10 image runs.

The stimuli included 5254 images, 4916 of which were unique. The images were obtained from three computer vision datasets: Scene UNderstanding (SUN) [21], Common Objects in Context (COCO) [22], and ImageNet [23]. They were downsampled to 375 × 375 pixels and subtended a visual angle of approximately 4.6 degrees. The stimuli were presented using an event-related design. Each run comprised 37 stimuli, with approximately 2 from repeated images. Each image was presented for 1 second followed by a fixation cross for 9 seconds. At the beginning and end of each run, a fixation cross was displayed for 6 seconds and 12 seconds, respectively. fMRI data were acquired using a 3 T Siemens Verio MR scanner at the Carnegie Mellon University campus with a 32-channel phased array head coil. The repetition time (TR) was 2000 ms, the echo time (TE) was 30 ms, the field of view was 212 mm, and the slice thickness was 2 mm.

The data we used covered five visual areas in the human visual cortex, i.e., early visual area (EarlyVis), the lateral occipital complex (LOC), the occipital place area (OPA), the parahippocampal place area (PPA), and the retrosplenial complex (RSC). Note that different visual areas perform different visual functions. EarlyVis in this dataset goes beyond the typical V1 and V2 areas. Human visual cortex V1 is mainly responsible for the detection of local features and provides this information to the middle or even higher visual areas [24, 25]. V2 has a slightly complex modulation for positioning, spatial frequency color, and moderate modulation for complex shape [26, 27]. The other four areas belong to advanced visual areas, which perform more complex visual tasks such as perceiving the boundaries of a scene [28], processing shape [29], encoding and recognizing an environmental scene [30], and dealing with scenarios [31].

### 2.2. Overview of the Proposed Method

**Figure 1:**
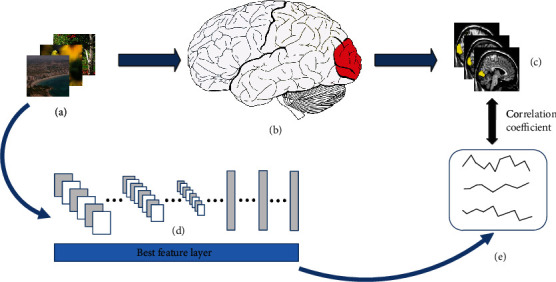
Main process of visual encoding. (a) Natural image stimuli; (b) visual processing of human brain; (c) real fMRI responses obtained by an MRI scanner; (d) CNN features of natural images extracted by pretrained CNN; (e) predicted voxel responses. When subjects saw the visual stimuli, the corresponding brain signals would be generated in the visual areas of the brain, and the fMRI responses were obtained through the MRI scanner. Using the pretrained network to extract the features of natural images, the CNN features of each layer were linearly mapped to voxel space, and the feature layer with the best prediction performance was selected as the best encoding feature layer to obtain predicted voxel responses. Then, the correlation coefficient between predicted responses and real responses was calculated to evaluate the prediction performance of the encoding model.

In general, linearized encoding adopts a two-step strategy, requiring two computational models to encode voxels. The first one is feature transformation, which is a nonlinear mapping from input space to feature space using feature extractors. The other is a linear regression model, which is a linear mapping from feature space to voxel space. The parameters of the feature transformation model are typically fixed and do not need further training. On the other hand, the linear weights of the linear regression model need to be trained. In this paper, we constructed CNN-based visual encoding models that use the classification network VGG and the segmentation network FCN to extract features of the input stimuli.  Figure 1 shows the overall process.

5254 natural images were randomly divided into a group of 4754 images and a group of 500 images. Two groups of the images and their corresponding voxel responses were considered, with one group used as the training set and the other as the test set. We employed pretrained VGG16 and FCN32s to accomplish feature transformation and then used ROMP to construct a linear regression model. We mapped the features extracted by the two networks and the fused features to the voxel responses of visual areas to learn the weight coefficients. Hence, we attained three encoding models based on different CNN features. The encoding models were then tested on the test set to obtain the prediction accuracy for each voxel. Here, we defined the prediction accuracy as the Pearson Correlation Coefficient between the observed and predicted responses across the test set. A high correlation coefficient corresponds to a high prediction accuracy of the encoding model, which means that the features and voxel responses are more linearly related.

### 2.3. Extracting Hierarchical Visual Features Based on VGG16

To extract the features of natural images using a classification network, we employed the pretrained model of VGG16 based on the open-source deep learning framework of PyTorch [32]. VGG16, which is a classification model proposed by Oxford University in 2014 [33], comprises of 16 hidden layers (13 convolutional layers and 3 fully connected layers). Each artificial neuron in the convolutional layer corresponds to a feature detector, called a feature map, which represents the characteristics of the input stimuli. Each convolutional layer has 64, 64, 128, 128, 256, 256, 256, 256, 512, 512, 512, 512, 512, 512, 512, and 1000 (class of the dataset) kernels. In order to make the gradient descent and reverse propagation more effective, the activation function called the Rectified Linear Unit (ReLU) [34] is used by the artificial neurons at layers 1–15. The pooling layer that reduces redundancy can also be interpreted as a form of the nonlinear downsampling operation. The most common form of pooling layer is maximum pooling and average pooling. In the VGG16 architecture, layers 2, 4, 7, 10, and 13 adopt maximum pooling, while layers 14 and 15 adopt a nonlinear transformation to eliminate regularization. The architecture of VGG16 is shown in Table 1.

### 2.4. Extracting Hierarchical Visual Features Based on FCN32s

To extract the features of natural images using a segmentation network, we employed pretrained FCN32s for semantic segmentation [11]. In this structure, the FCN converts fully connected layers into convolutional layers. The output image of the last layer is sampled 32 times to obtain the image with the same size as the original input image. FCN32s initializes the network with the structural parameters of VGG16, discards the final classification layer, and converts all fully connected layers into convolutional layers; hence, it is called a fully convolutional network. We used a 1 × 1 convolution with a channel size of 21 to predict the score of each location (including background) of the Pascal class. Then, the deconvolution layer was added to the output at the pixel level to sample the output upwards.

FCN32 comprises 16 convolutional layers with each having 64, 64, 128, 128, 256, 256, 256, 256, 512, 512, 512, 512, 512, 512, 4096, 4096, and 21 (class of the dataset) kernels. In the architecture of FCN32s, ReLu is used in layers 1–16. Layers 2, 4, 7, 10, and 13 adopt maximum pooling, while layers 14 and 15 adopt dropout regularization to realize nonlinear transformation. The architecture of FCN32s is shown in Table 1.

The FCN32s architecture we employed released in 2017 is available at https://github.com/meetshah1995/pytorch-semseg. We trained the FCN32s on 2913 high-resolution images from the Pascal-VOC 2012 dataset for semantic segmentation using PyTorch [32]. Each input image was represented as three RGB color channels and filtered through the convolutional layers. The stride of the convolutional layers was 3 pixels at layers 1–13, 7 pixels at layer 14, 1 pixel at layer 15, and 21 pixels at layer 16. In the training process, we adopted momentum and weight attenuation for random gradient descent. The learning rate was initialized to 0.01, and the final intersection over union (IOU) was 0.59. We trained FCN32s on the segmentation dataset to obtain a pretrained network for feature extraction of the encoding model.

### 2.5. Training the Mapping from the Features to Voxel Responses Based on Sparse Representation

Corresponding to each layer of the CNN features, a linear model can be constructed to map CNN features into voxel responses of the visual areas. For responses of one voxel to all training samples, the model can be expressed by

(1) $$\begin{array}{c} y=Xw+\varepsilon . \end{array}$$

Here, *y* is the measured voxel responses represented by an *m* − *by* − 1 matrix, where *m* is the number of training samples; *X* is the CNN features of images represented by an *m* − *by* − (*n* + 1) matrix, where *n* is the dimension of features and the last column is the constant vector; *w* is the weight coefficient to be solved represented by an (*n* + 1) − *by* − 1 matrix; and *ε* is the noise term.

However, the number of training samples *m* is significantly smaller than the number of voxels *n* in visual areas. Hence, Equation (1) is an ill-posed equation without a unique solution. In addition, in several studies [35, 36], the visual cortex uses sparse coding for the expression of stimuli, which means that a specific stimulus can only activate a few specific visual neurons. Hence, sparse representation can be used as an effective tool to encode information related to natural images. Considering a sparse coefficient *w*, Equation (1) is converted into a traditional sparse representation problem, which is typically considered as an NP-Hard problem, defined as follows:

(2) $$\begin{array}{c} \underset{w}{\min}{\left\|w\right\|}_{0}\text{ subject to }Xw=y. \end{array}$$

To approximate the solution of Equation (2), we used the greedy algorithm [37], which follows the heuristic of making the locally optimal choice at each stage with the intent of finding a global optimum, which is quite fast by computing the support of the sparse signal iteratively [38]. Considering that the encoding model must be estimated for each voxel, the method we need to employ should be fast enough and simple to reduce the time cost. Therefore, we used the greedy algorithm to investigate the sparseness of the encoding model, in particular, the ROMP algorithm.

ROMP is an iterative fitting technique that reduces the difference between model fit and data [39, 40]. The specific calculation process is shown in Algorithm 1.

The features of each layer in FCN32 and VGG16 on the training set were mapped to the voxel space by ROMP, and the weight coefficients were obtained. Here, the coefficient of the final nonzero term was 100. Then, the predicted voxel responses for the test set were obtained through the weight coefficients of each layer. We compared the correlation between the predicted responses and the measured voxel responses. Based on the correlation coefficient, the highest prediction accuracy was selected for each voxel; that is, the feature layer with the highest correlation was taken as the best feature layer for each voxel. The linear mapping from the best feature layer to the voxel response was added to obtain the voxel-wise encoding model.

### 2.6. Combined Encoding Model Based on the Fusion of Features

To fit the diversity of the mechanism of human vision, we fused some image features extracted from the classification and segmentation networks and established an encoding model based on the fused features. Firstly, we employed the ROMP algorithm to construct a linear mapping from the voxel responses to all the image features extracted from the FCN32s and VGG16 on the training set and obtained the predicted image features on the test set. For the specific one-dimensional feature on a certain layer of CNN, the model can be expressed by

(3) $$\begin{array}{c} y_{1}=X_{1}w_{1}+\varepsilon_{1}. \end{array}$$

Here, *y*_1_ is the CNN features of images represented by an *p* − *by* − 1 matrix, where *p* is the number of training samples; *X*_1_ is the measured voxel responses represented by an *p* − *by* − (*q* + 1) matrix, where *q* is the number of voxels and the last column is the constant vector; *w*_1_ is the weight coefficient to be solved represented by a (*q* + 1) − *by* − 1 matrix; and *ε*_1_ is the noise term.

To reduce the influence of ineffective features, we calculated correlation coefficients between the predicted and real image features. According to the ranking of correlation coefficients from largest to smallest, the corresponding image features of the first 10% dimension (including the part of image features extracted by FCN32s and VGG16) were selected at each layer.

After feature selection, visual encoding was carried out according to the method mentioned in Training the Mapping from the Features to Voxel Responses Based on Sparse Representation. The features of selected dimensions were extracted from the training set and linearly mapped to the voxel responses by ROMP. Then, the predicted voxel responses based on different feature layers (including image features extracted from FCN32s and VGG16) were obtained by using the calculated weights. For each voxel, the feature with the highest prediction accuracy was selected as the best feature layer, and the voxel-wise visual encoding model was established.

### 2.7. Quantitative Standards

We define the prediction accuracy for a voxel as a Pearson Correlation Coefficient between the measured and the predicted responses across all 500 images in the test set:

(4) $$\begin{array}{c} r=\frac{\mathrm{cov}\left(v_{\text{p}},v_{\text{m}}\right)}{\sqrt{\mathrm{var}\left[v_{\text{p}}\right]\left[v_{\text{m}}\right]}}. \end{array}$$

In Equation (4), *v*_p_ represents the predicted voxel responses, *v*_m_ represents the measured voxel responses in the test set, and *r* represents the correlation coefficient between them, i.e., the encoding accuracy.

To examine whether each voxel's prediction accuracy value significantly deviated from the null hypotheses, we randomly shuffled the pairing between measured and predicted responses across 500 images in the test set 1000 times and in each randomized sample recalculated the voxel's prediction. This calculation constructed a null hypothesis distribution for each voxel. For all voxels, the prediction accuracy value above 0.13 was significant (*p* < 0.001) relative to its null hypothesis distribution.

To examine the significance of a model advantage, that is, the number of voxels that can be predicted by the model that is significantly more than that of the other, we randomly permuted (with a probability of 50%) the prediction accuracy of each voxel of the two models being compared and then calculated the advantage of each model (the percentage of voxels with the highest prediction accuracy). In this paper, we repeated such permutations 1000 times, and null hypothesis distribution was obtained. From the null hypothesis distribution, it is concluded that for any two models, the model which can accurately predict more than 53% of voxel responses is significantly better than the other model (*p* < 0.05).

## 3. Results

### 3.1. Comparison of Prediction Accuracy

#### 3.1.1. Comparison of VGG16-Based and FCN32-Based Encoding Models

**Figure 2:**
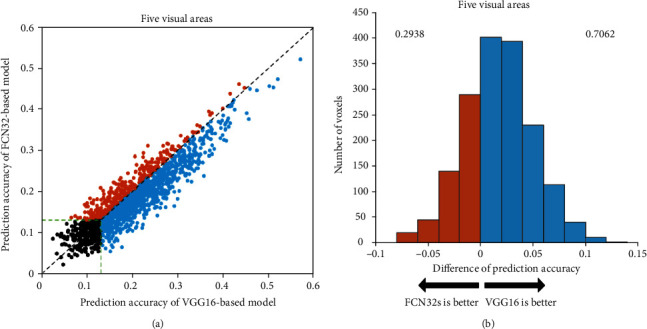
Comparison of the prediction accuracy between FCN32-based and VGG16-based encoding models in five visual areas of subject 1. (a) Prediction accuracies. The abscissa and ordinate represent the prediction accuracy of the FCN32-based encoding model and the VGG16-based encoding model, respectively. The orange dots represent the voxels that can be better predicted by the FCN32-based model than the VGG16-based model. The blue dots represent the opposite. And the black dots represent voxels with prediction accuracy less than 0.13. The green dashed lines indicate that the prediction accuracy is 0.13. (b) Distribution of the difference in prediction accuracies. The blue color denotes that the prediction accuracy is higher for the VGG16-based model. The orange color denotes that the prediction accuracy is higher for the FCN32-based model. The numbers on each side indicate the fraction of voxels with higher prediction accuracy under the model.

**Figure 3:**
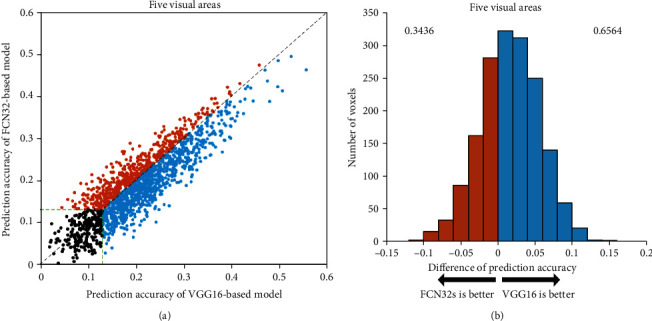
Comparison of prediction accuracy between FCN32-based and VGG16-based models for subject 2. Refer to Figure 2 for a detailed description of the plot elements.

**Figure 4:**
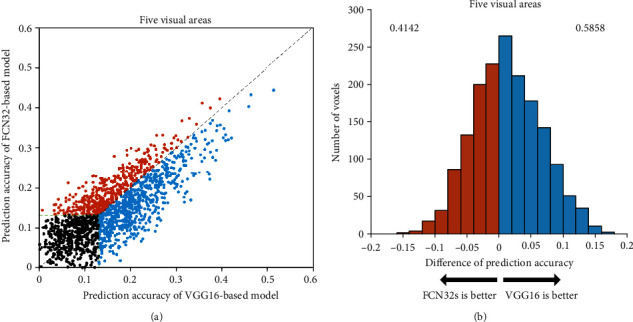
Comparison of prediction accuracy between VGG16-based and FCN32-based models for subject 3. Refer to Figure 2 for a detailed description of the plot elements.

To evaluate the encoding capabilities of different networks based on different training tasks, we calculated the prediction accuracy of voxels in five ROIs based on two encoding models: classification network and segmentation network. We used a scatter plot to compare the accuracy of the two models and analyze their performances. Each plot represents a single voxel from the five ROIs. The ordinate of each point represents the highest encoding accuracy of the FCN32s model, while the abscissa represents the highest encoding accuracy of the VGG16 model. Here, the correlation threshold for significance prediction is 0.13 (*p* < 0.001). The results show that the prediction accuracy of the encoding model based on VGG16 is better than that of the encoding model based on FCN32s.  Figure 2 show the results for subject 1, while the results for subjects 2 and 3 are presented in Figures 3 and 4.

The VGG16-based model has significant advantages over the FCN32-based model in the five visual areas (*p* < 0.05). The results show that the encoding performance of the network based on classification features is significantly better than that of the network based on segmentation features, which indicates that different network tasks can affect the performance of the encoding model. However, some voxels have better prediction accuracy in the FCN32-based model than in the VGG16-based model, which indicates that there are still inconsistencies between the classification or segmentation networks and the visual encoding mechanism of the human brain.

#### 3.1.2. Comparison between VGG16-Based, FCN32-Based, and Fused Feature-Based Encoding Models

**Figure 5:**
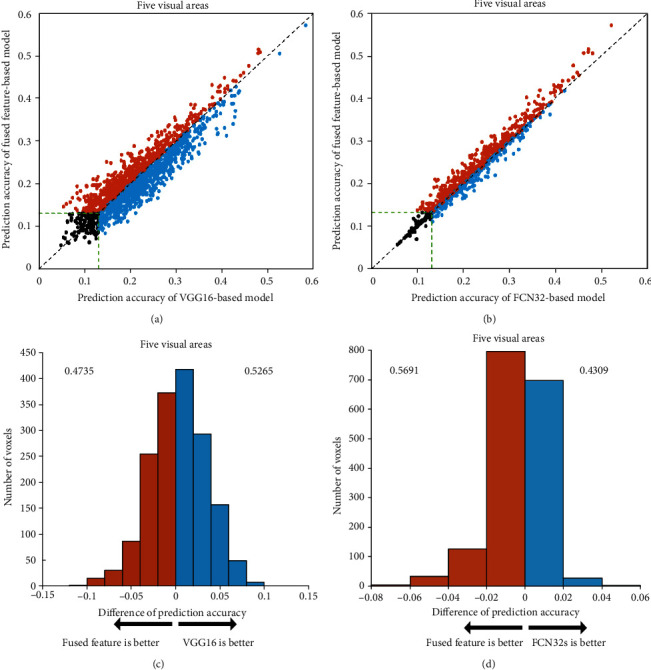
Comparison of the prediction accuracy between the fused feature-based encoding model and (a) VGG16-based or (b) FCN32-based encoding models in five visual areas. The ordinate represents the prediction accuracy of the fused feature-based encoding model, and the abscissas represent the prediction accuracy of the VGG16-based or FCN32-based encoding models. The orange dots represent the voxels that can be better predicted by the fused feature-based model than the VGG16-based or FCN32-based models. The blue dots represent the opposite. The green dashed lines and the black dots represent the same meanings as Figure 2. (c) Distribution of the difference between fused features and VGG16 or (d) FCN32-based model in prediction accuracies. The blue color denotes that the prediction accuracy is higher for the VGG16-based model or FCN32-based model. The orange color denotes that the prediction accuracy is higher for the fused feature-based model. The numbers on each side indicate the fraction of voxels with higher prediction accuracy under the model.

To explore the relationship between segmentation features and classification features in visual encoding, i.e., the intersection and union of classification and segmentation tasks in the human visual system, we compared the prediction performance of the encoding model based on fused features with that of the VGG16-based and FCN32-based encoding models. The results shown in Figure 5 are used to compare the accuracy of the three models and analyze their performances.

**Figure 6:**
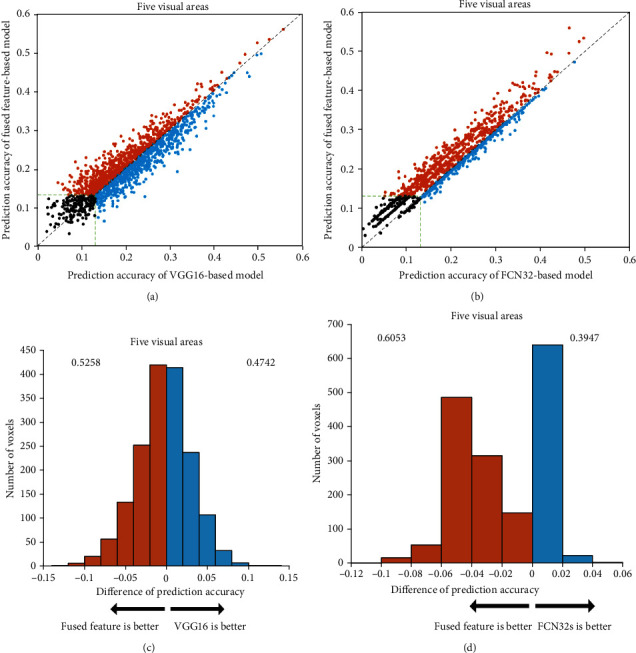
Comparison of prediction accuracy between fused feature-based and VGG16-based or FCN32-based models for subject 2. Refer to Figure 5 for a detailed description of the plot elements.

**Figure 7:**
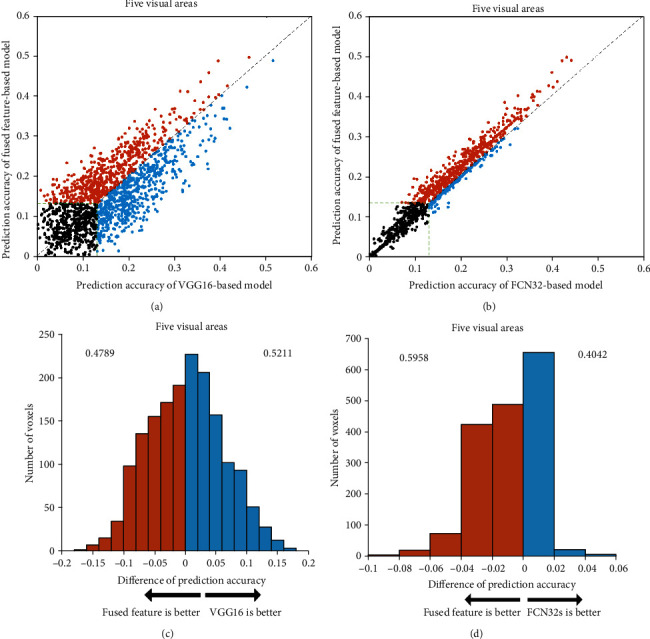
Comparison of prediction accuracy between fused feature-based and VGG16-based or FCN32-based models for subject 3. Refer to Figure 5 for a detailed description of the plot elements.

Consistent with the results of subject 2 and subject 3 in Figures 6 and 7, the prediction performance of the fused feature-based encoding model is significantly better than that of the FCN32-based encoding model (*p* < 0.05), while it is slightly different from that of the VGG16-based encoding model. To a certain extent, this indicates that the fused features can significantly improve the prediction performance of the encoding model compared with the segmentation features but have little effect compared with the classification features. In other words, in the process of the human visual system perceiving external stimuli, the classification task performed by the visual areas covers most of the segmentation task; that is, in the process of completing the classification of external objects, the segmentation of objects is basically completed, which means that people can recognize the category, size, and location of objects almost at the same time when they see a picture.

### 3.2. Relationship between Feature Quantity and Prediction Accuracy

**Figure 8:**
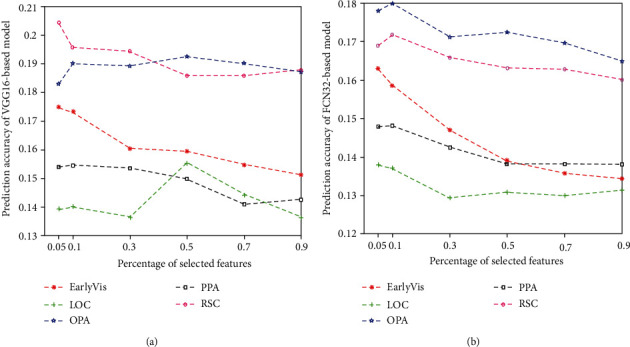
Relationship between the percentage of selected features and prediction accuracy of two encoding models in five visual areas: (a) VGG-based model and (b) FCN32-based model. The abscissa represents the percentage of selected features, and the ordinate represents the prediction accuracy of the models. The lines represent the results for five different visual areas.

**Figure 9:**
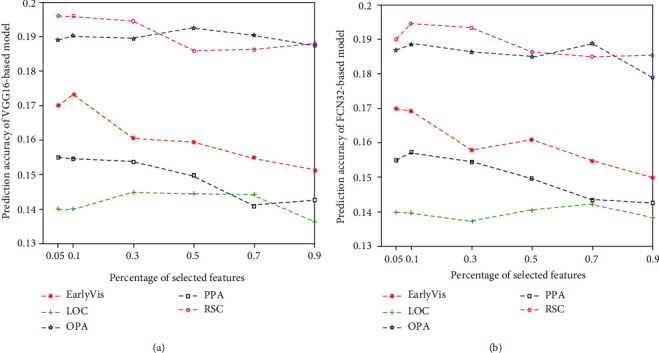
Relationship between the percentage of selected features and prediction accuracy of two encoding models (VGG16-based and FCN32-based models) in five visual areas of subject 2. Refer to Figure 8 for a detailed description of the plot elements.

**Figure 10:**
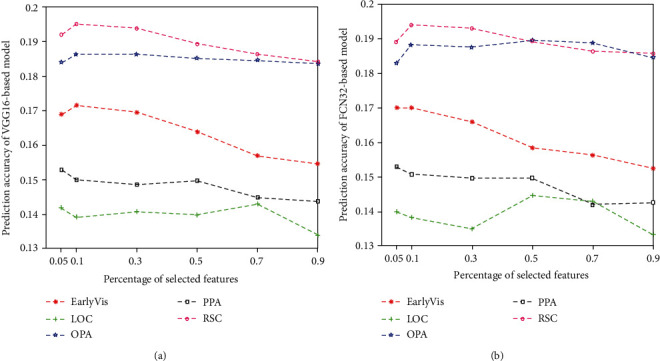
Relationship between the percentage of selected features and prediction accuracy of two encoding models (VGG16-based and FCN32-based models) in five visual areas of subject 3. Refer to Figure 8 for a detailed description of the plot elements.

We compared and analyzed the influence of the number of features on the encoding performance for subject 1, as shown in Figure 8. Results for subject 2 and subject 3 are shown in Figures 9 and 10. The results show that too few or too many features can negatively impact the performance of the encoding model. In particular, a small number of features lead to the lack of effective information, while a high number of features lead to redundancy of effective information.

### 3.3. Contribution of Each CNN Layer to Prediction Performance

**Figure 11:**
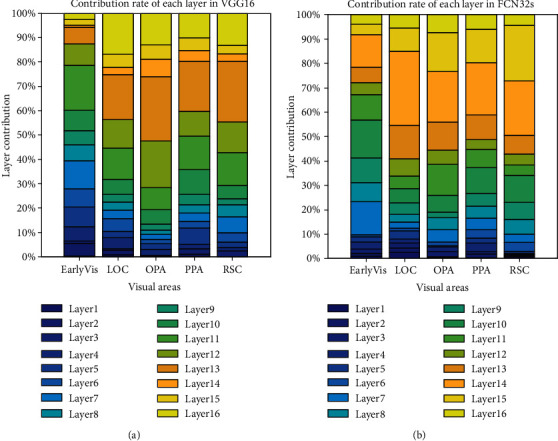
Contribution to the prediction accuracy of each layer in (a) VGG16 and (b) FCN32 networks. The ordinate represents the contribution of each layer, and the abscissa represents the five visual areas. The color bar from deep to shallow indicates the network layer from a low level to a high level.

**Figure 12:**
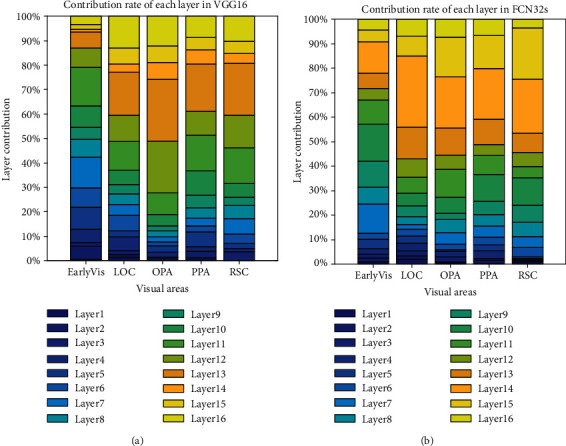
Contribution to the prediction accuracy of each layer in (a) VGG16 and (b) FCN32s networks for subject 2. Refer to Figure 11 for a detailed description of the plot elements.

**Figure 13:**
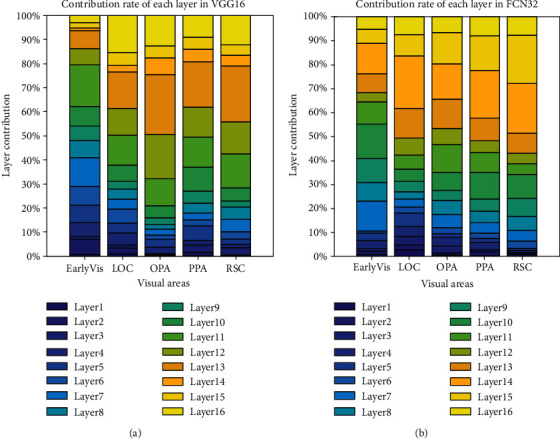
Contribution to the prediction accuracy of each layer in (a) VGG16 and (b) FCN32 networks for subject 3. Refer to Figure 11 for a detailed description of the plot elements.

To further compare the encoding differences of different networks based on different training tasks as feature models and verify the hierarchical similarity between CNNs and the human visual system, we analyzed the best encoding feature layer of the two CNNs. In detail, for voxels of different ROIs, we counted which layer of the CNN the best encoding layer came from.  Figure 11 shows the contribution of each layer of the two feature models to voxel responses in different visual areas. And the results of subject 2 and subject 3 are shown in Figures 12 and 13. From the figures, it is clear that voxel responses of the primary visual area can be better predicted by features in lower-level layers irrespective of the network's task. For the other four high-level visual areas, features in higher-level layers can better predict voxel responses (Mann-Kendall method, *p* < 0.05).

This study verifies the hierarchical similarity between CNNs and the human visual system. It also confirms that the human visual system and CNNs similarly process visual information in a hierarchical manner [15, 16]. Specifically, in the visual information processing pathway of the human brain, primary visual areas process relatively simple information, such as edges and shapes, and advanced visual areas process more complex visual features such as semantics and color. This is similar in CNNs where lower layers deal with simpler features and deeper layers deal with more complex features.

## 4. Discussions

### 4.1. Encoding Model Based on the Classification Network (VGG16) Has Better Prediction Performance

From the obtained prediction accuracies, we found that the encoding model based on the classification network is superior to that based on the segmentation network. Meanwhile, the prediction performance of the encoding model based on fused features is significantly better than that of the model based on segmentation and is almost the same as that of the model based on classification.

Our results show that different networks based on different computer vision tasks can affect the performance of the encoding models. We can also infer, to some extent, that the visual classification task can better fit human visual information processing than the visual segmentation task, with the human brain already completing the segmentation of objects in the process of completing the visual classification task. This is consistent with the discovery of David H. Hubel and Torsten Wiesel, 1981 Nobel Prize winners, that the information processing of the visual system is hierarchical in visual areas and the working process of the brain is iterative and abstract [41]. Upon obtaining the original information by the retina, visual area V1 firstly processes features related to edges and directions. Then, visual area V2 processes features related to contours and shapes. Finally, higher visual areas perform more refined classifications through more high-level abstractions iteratively. Hence, the human visual system already implements most of the segmentation tasks during information processing to realize the classification of external stimuli. This process is embedded in our brain and happens almost instantaneously.

From the point of view of natural evolution, primitive humans only need to identify whether an object in the field of vision is threatening them to avoid risk. This means that the object only needs to be categorized without the need for a specific segmentation.

From the experimental point of view, the subjects performed a task to judge the likes and dislikes of the input stimuli, which does not involve specific visual segmentation. This may have limitations that could affect the encoding performance. However, we can deduce that in the case of humans performing default visual tasks, the visual system gives priority to the classification of objects. On the other hand, when performing specific visual tasks, such as visual attention tasks, humans may give more priority to object segmentation.

### 4.2. Relationship between Classification and Segmentation Tasks in the Human Visual System

The visual encoding model based on classification and segmentation task-driven networks has advantages in predicting voxel responses, which indicates that the human visual system cannot be completely simulated by a certain task-driven network and performs various and complex visual tasks during visual information processing. We found that the prediction performance of the encoding model based on classification features is significantly better than that of the model based on segmentation features; hence, the CNN performing the classification task is more similar to the human visual system. The encoding model based on fused features and that based on classification features have almost the same performance, which indicates that the classification task is similar to most of the segmentation task. In other words, during visual processing, the human brain completes most of the visual segmentation when the visual stimuli are classified.

### 4.3. The Prediction Accuracies of the Three Models Are Not High

From the perspective of encoding efficiency, Güçlü and van Gerven [16] employed a motion recognition network to predict the voxel responses in the dorsal pathway. In addition, a recent study investigated the impact of different computer vision tasks on deep networks performing visual encoding [19]. This demonstrates that research on encoding efficiency is beginning to gain attraction in the field.

In this study, we used the BOLD5000 dataset, which is the largest publicly published dataset. However, the obtained prediction accuracies of the three encoding models are not particularly high, which may be related to the diversity of stimuli in the dataset and absence of restrictions of the subjects' sights in the experiments. It should be emphasized that subjects only judged whether they liked or disliked the input images during the experiment. Hence, this limitation in the task may have an impact on the encoding performance. Moreover, it is unknown whether the performance of the encoding model based on the segmentation network would be improved if the subjects performed a corresponding visual segmentation task. This needs to be addressed in future work, highlighting its importance and relevance.

## 5. Conclusions

In conclusion, we explored the impact of different networks based on different tasks on encoding models. We found that the performance of the encoding model based on fused features is significantly better than that of the model based on segmentation and is almost the same as that of the model based on classification. This demonstrates that the CNN performing the classification task is more similar to the human visual system, and most of the segmentation of the visual system for the stimuli is completed with the process of object classification. However, we also found that the encoding model based on segmentation had better prediction performance on some voxels, which further illustrates the complexity and diversity of the human visual mechanism. In the future, we will consider more types of networks that perform different computer vision tasks, such as target detection and object recognition, which are aimed at not only improving the prediction performance but also better realizing the mechanism of human vision. Here, we demonstrated a valuable way of developing a computational neuroscience model from the perspective of computer vision.

## Figures and Tables

**Algorithm 1:**
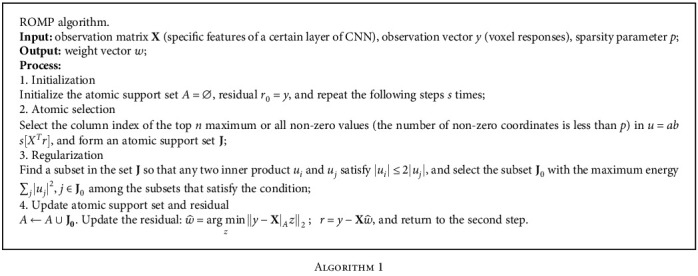


**Table 1:** The layer index and corresponding layer names of VGG16 and FCN32s.

Index	1	2	3	4	5	6	7	8
Layer name of VGG16	conv1	conv2 mpool	conv3	conv4 mpool	conv5	conv6	conv7 mpool	conv8
Layer name of FCN32s	conv1	conv2 mpool	conv3	conv4 mpool	conv5	conv6	conv7 mpool	conv8
Index	9	10	11	12	13	14	15	16
Layer name of VGG16	conv9	conv10 mpool	conv11	conv12	conv13 mpool	fc1	fc2	fc3
Layer name of FCN32s	conv9	conv10 mpool	conv11	conv12	conv13 mpool	conv14	conv15	conv16

## Data Availability

The data used to support the findings of this study are available from the corresponding author upon request.
